# Increased national critical care demands were associated with a higher mortality of intubated COVID-19 patients in Japan: a retrospective observational study

**DOI:** 10.1186/s40560-024-00758-8

**Published:** 2024-11-05

**Authors:** Kazuya Kikutani, Mitsuaki Nishikimi, Ryo Emoto, Shigeyuki Matsui, Hiroyuki Ohbe, Takayuki Ogura, Satoru Hashimoto, Shigeki Kushimoto, Shinhiro Takeda, Shinichiro Ohshimo, Nobuaki Shime

**Affiliations:** 1 Department of Emergency and Critical Care Medicine, Graduate School of Biomedical and Health Sciences, Hiroshima University, 1-2-3 Kasumi, Minami-ku, Hiroshima, 734-8551 Japan; 2 Department of Biostatistics, Nagoya University Graduate School of Medicine, Nagoya, Japan; 3 Department of Emergency and Critical Care Medicine, Tohoku University Hospital, Sendai, Japan; 4 Non-Profit Organization Japan ECMO Network, Tokyo, Japan; 5 Non-Profit Organization ICU Collaboration Network (ICON), Tokyo, Japan

**Keywords:** COVID-19, Respiratory failure, Mechanical ventilation, Surge capacity

## Abstract

**Background:**

There was no study to investigate the association between the national surge of Coronavirus disease 2019 (COVID-19) patients and the mortality of mechanically ventilated COVID-19 patients. The aim of this study was to assess the association between mortality in mechanically ventilated COVID-19 patients and two distinct national COVID-19 surge indices: (1) the daily number of newly confirmed COVID-19 cases, representing overall medical demands and (2) the total number of critically ill COVID-19 patients, reflecting critical care demands.

**Methods:**

We analyzed the patient data registered in a national database of mechanically ventilated COVID-19 patients between February 6, 2020, and May 16, 2023, combined with the data officially published by the Japanese government. Multivariable logistic regression analysis was performed to evaluate the association of these two indices with COVID-19 mortality. A generalized linear mixed effect model was used to examine the relationships between the variation in the impact of critical care demands across hospitals and the variation in baseline risk across hospitals.

**Results:**

The data of 8327 patients from 264 centers in Japan were analyzed. The overall mortality rate was 24% (1990/8327). The critical care demands, but not overall medical demands, were independently associated with the mortality (OR, 1.11; 95% CI 1.07–1.16; *p* < 0.001). This effect of critical care demands on the mortality was more pronounced in hospitals with higher baseline risk (*r* = 0.67).

**Conclusions:**

The national critical care demands were independently associated with the mortality of COVID-19 patients requiring mechanical ventilation. This effect was more pronounced in hospitals with higher baseline risk.

**Supplementary Information:**

The online version contains supplementary material available at 10.1186/s40560-024-00758-8.

## Background

Coronavirus disease 2019 (COVID-19), first identified in Wuhan, China, in December 2019, spread rapidly worldwide to cause a large number of deaths [1]. During the COVID-19 pandemic, approximately 4–12% of patients needed mechanical ventilation [2, 3], and were treated by intensive care specialists. COVID-19 caused fluctuations in the demands and depletion of medical resources, such as mechanical ventilators, intensive care unit (ICU) beds, etc., in intensive care units (ICUs) around the world [4]. The medical resources, such as ICU beds, ventilators, and medical staff, are crucial for the survival of critically ill patients. Several studies have shown that the increased demands and shortage of medical resources were associated with a higher mortality, not only among general ICU patients [5, 6] but also specifically in mechanically ventilated COVID-19 patients [7–12]. This may be likely due to the inability to provide timely and appropriate treatment. For example, COVID-19 often required medical resources for intensive care like ventilators and ECMO [13], and resulted in shortage of these resources. The resource shortages can lead to a decline in care quality and delays in critical interventions, both of which may significantly impact patient outcomes. Therefore, monitoring medical demand through specific indices could help predict the increased risk of mortality in severe COVID-19 cases.

Among the several indices reflecting the demands for medical resources, the nationwide number of COVID-19 patients is a straightforward one, with many countries accurately estimating the number of COVID-19 in their own countries during the pandemic, to assess the overall level of demand for healthcare resources in a country [14]. Because previous studies showed a significant association between medical resource demands at hospital level and patient mortality, we hypothesized that increased medical resource demands at the country level might be also associated with increased mortality of COVID-19 patients requiring mechanical ventilation.

The objective of this study was to investigate the association between the mortality of mechanically ventilated COVID-19 patients and two COVID-19 surge indices determined from the national database officially published by the Japanese government. These indices are: (1) the overall medical demands, which solely refer to the number of newly confirmed COVID-19 patients nationwide per day and (2) the critical care demands, which represent the total number of severe COVID-19 patients nationwide (defined as patients under mechanical ventilation or in the ICU). These indices were matched to the intubation date of each patient. Furthermore, we explored whether the variation in the impact of the critical care demands on the mortality of severe COVID-19 patients across hospitals correlates with the variation in baseline risk across hospitals.

## Methods

### Study design and data collection

This population-based retrospective cohort study targeting all citizens of Japan was conducted using data from the CRoss Icu Searchable Information System (CRISIS), the largest national database of severe COVID-19 patients in Japan (UMIN000041450) [15], combined with data officially published by the Japanese Ministry of Health, Labour and Welfare (MHLW) [16, 17]. CRISIS was developed by the Japan extracorporeal membrane oxygenation network (Japan ECMOnet) for COVID-19 to track real-time information from ICUs across Japan during the COVID-19 pandemic [15]. The CRISIS database covers more than 6600 ICU beds, equivalent to approximately 90% of all ICU beds in Japan [18].

Information on all COVID-19 patients requiring mechanical ventilation or extracorporeal membrane oxygenation (ECMO) from February 2020 at all participating hospitals was collected by CRISIS. The CRISIS data included the patient age, sex, body mass index (BMI), need for ECMO, respiratory support adopted prior to tracheal intubation, date of start of mechanical ventilation, and patient outcome [15]. All the patients registered in the CRISIS database were followed up until death, hospital transfer, or hospital discharge [19]. The primary outcome was mortality during the follow-up period. This study was conducted with the approval of the Hiroshima University Epidemiological Research Ethics Review Committee (approval numbers E2022-0118), which waived the requirement for obtaining informed consent from the study participants to ensure participant anonymity, as stipulated in the Japanese government guidelines.

### Inclusion and exclusion criteria

Mechanically ventilated patients registered in the CRISIS database who were initiated on mechanical ventilation between February 6, 2020, and May 16, 2023 (i.e., until the 8th wave of the COVID-19 epidemic in Japan) were eligible for inclusion in the study. The exclusion criteria were patients younger than 18 years of age, those who were transferred to another hospital during the acute phase (i.e., when they remained intubated without tracheostomy or potentially needed ECMO), those with missing data required for the main analysis, and those admitted during the first wave of the COVID-19 pandemic in Japan, because the Japanese MHLW had started to count and officially announce the number of severe COVID-19 patients several weeks before the start of the 2nd wave. The period of each wave was determined based on the definition reported in an official Public Health website in Japan [20].

### Definition of the national surge indices for COVID-19

The following two COVID-19 surge indices at the country level were determined for each analyzed subject: (1) the number of newly confirmed COVID-19 patients nationwide per day (overall medical demands), and (2) the total number of severe COVID-19 patients nationwide (defined as patients under mechanical ventilation or in the ICU) (critical care demands). These indices were corresponded to the intubation date of each patient, as in our previous study [10]. Both indices, used in several studies as reliable indices for evaluating the epidemic waves in Japan [21, 22], were obtained from the national database officially published by the Japanese MHLW [16]. In order to transform the unit of these indices from number to percentage, the numbers were divided by the total number of hospital beds designated for COVID-19 patients in Japan at the beginning of the study period and the number of ICU beds for COVID-19 in Japan at the beginning of the study period (19,474 beds and 2535 beds on June 10, 2020) [17], respectively.

### Statistical analysis

Continuous variables are represented as medians (interquartile range) and categorical variables as *n* (%). Multivariable logistic regression analysis was performed using the following variables for adjustment; age, sex, BMI, use of ECMO (±), use of non-invasive ventilation (NIV) or high-flow nasal cannula (HFNC) before intubation (±), and the wave of the COVID-19 epidemic; in addition to variables related to basic patient information and disease severity, we decided to add the epidemic wave number as a variable for adjustment, because the mortality rates varied among the different waves of the epidemic [23]. We treated BMI as a categorical variable. Since the number of patients classified as underweight (BMI below 18.5) was very small, we combined this group with those with BMI less than 25. Therefore, BMI was categorized into groups: less than 25, 25 or more but less than 30, and 30 or more. Multivariable analysis was performed separately for the overall medical demands and critical care demands, because we considered these two indices showed multicollinearity. We also performed sensitivity analysis using a generalized linear mixed-effects model considering the variation in the baseline risk across hospitals. We also fitted a multivariable logistic regression model with a thin plate spline for critical care demands using the same prognostic variables for adjustment. Furthermore, as an additional sensitivity analysis, we conducted a multivariable logistic regression analysis including the hospital-level congestion index (hospital level demands) as an adjustment variable. The hospital level demands was defined as average number of other COVID-19 patients on ventilator management at the facility during the period that a particular COVID-19 patient is on ventilator management.

To evaluate the correlation between variations in the impact of critical care demands and the baseline risk across hospitals, we fitted the model by Bayesian estimation with non-informative prior and calculated the Bayes factor for comparison of two models: one with independent random effects and the other with positively correlated random effects. We judged a value of Bayes factor of more than 50 as representing strong evidence [24].

We used the R package “mgcv” for the multivariable logistic regression model with a thin plate spline, “lme4” for the generalized linear mixed-effects model, and “brms” and “rstan” for Bayesian estimation and Bayes factor calculation, respectively. All analyses were performed using R 4.1.1.

## Results

### Patient characteristics

**Fig. 1:**
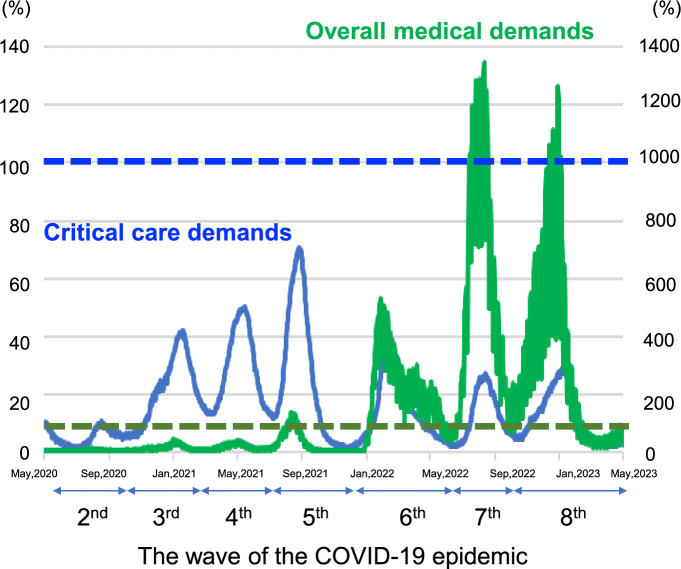
Trend of critical care demands and overall medical demands in Japan. The solid blue line represents the total number of severe COVID-19 patients divided by number of beds for severe COVID-19 patients × 100 (%) (critical care demands), and the solid green line represents the number of newly infected patients divided by the number of hospitalized beds for COVID-19 × 100 (%) (overall medical demands). The blue and green dotted lines represent 100% of each. This data was collected from the national database officially published by the Japanese government [16, 17]. During the study period, the following time frames were designated as epidemic waves based on Japanese public standards [20]: June 14, 2020 to October 9, 2020 was referred to as the “second wave,” October 10, 2020 to February 28, 2021 as the “third wave,” March 1, 2021 to June 20, 2021 as the “fourth wave,” June 21, 2021 to December 16, 2021 as the “fifth wave,” December 17, 2021 to June 24, 2022 as the “sixth wave,” June 25, 2022 to September 26, 2022 as the “seventh wave,” and September 27, 2022 onwards as the “eighth wave”

The trends in the number of newly confirmed COVID-19 patients and the number of severe COVID-19 patients across Japan during the study period are shown in Fig. 1. The patient flow into this study is shown in Supplementary Fig. 1. A total of 12,286 patients were screened from 322 CRISIS participating facilities. Of these, 997 patients were excluded due to outside the study period (*n* = 845), they were aged under 18 years (*n* = 138), cases where the initiation and end dates of mechanical ventilation were inconsistent (*n* = 14). An additional 2962 patients were excluded due to missing data, including those who had been transferred to another hospital during the acute phase (*n* = 338). Among the remain 2624 cases with missing data, 36 cases were missing gender, 35 cases were missing age, 918 cases were missing BMI, 1231 cases were missing HFNC or NPPV before intubation, and 404 cases were missing outcome (death). The remaining 8327 patients from 264 centers who were admitted between the 2nd and 8th waves of the COVID-19 epidemic in Japan were finally included in the analysis. The patient characteristics are shown in Table 1. The median age of the patients was 65 [54, 74] years, 74.6% (6213/8327) were male. Of these patients, 944 (11.3%) received ECMO. The overall mortality of the analyzed patients was 23.9% (1990/8327).

**Table 1:** Baseline characteristics of the patients with severe COVID-19

	*N* = 8327
Age, median [IQR]	65 [54, 74]
Sex, male, *n* (%)	6213 (74.6)
BMI	
BMI < 25	3923 (47.1)
25 ≤ BMI < 30	2792 (33.5)
BMI ≥ 30	1612 (19.4)
Use of ECMO, *n* (%)	944 (11.3)
Use of NIV or HFNC before MV, *n* (%)	2773 (33.3)
Pandemic wave	
Second wave, *n* (%)	506 (6.1)
Third wave, *n* (%)	2133 (25.6)
Fourth wave, *n* (%)	2202 (26.4)
Fifth wave, *n* (%)	2086 (25.1)
Sixth wave, *n* (%)	751 (9.0)
Seventh wave, *n* (%)	313 (3.8)
Eighth wave, *n* (%)	336 (4.0)
Mortality, *n* (%)	1990 (23.9)

*BMI* body mass index, *ECMO* extracorporeal membrane oxygenation, *NIV* non-invasive ventilation, *HFNC* high-flow nasal cannula, *MV* mechanical ventilation

### Association of the national medical demands for COVID-19 with the mortality

**Fig. 2:**
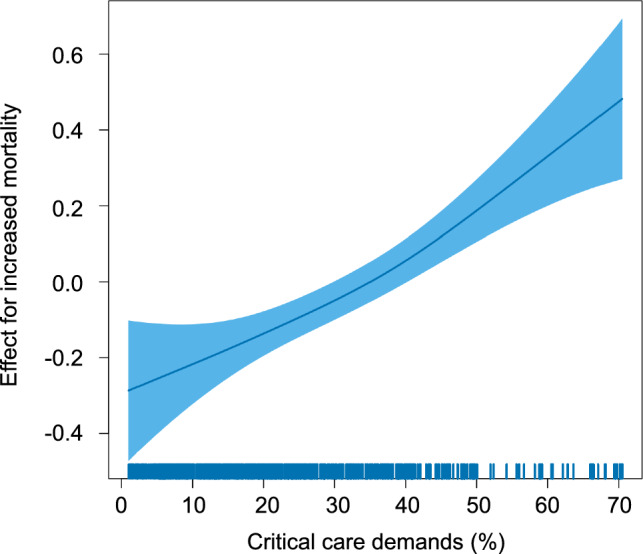
Spline curves showing the effect of critical care demands on mortality. The spline curves showed an almost linear effect of the critical care demands on the mortality. The horizontal axis represents critical care demands and the vertical axis represents the estimated values of the log odds ratio

Multivariable logistic regression analyses failed to reveal any association between the overall medical demands and the mortality (ORs: 1.00 [1.00–1.01, *p* = 0.63]) (Table 2), but an independent positive association between the critical care demands and the mortality (ORs: 1.11 [1.07–1.16, *p* < 0.001]) (Table 3). The ORs were also calculated for every 10% increase in the surge indices. We confirmed similar results in the sensitivity analysis, even when we added hospital-specific effect as a random effect in the generalized linear mixed-effects model (Supplementary Table 1). In order to further evaluate the relationship between the critical care demands and mortality, we also plotted spline curves, which showed an almost linear relationship (Fig. 2). As another sensitivity analysis, we additionally performed a generalized linear mixed-effects model by including hospital level demands as an adjustment factor, which can more directly reflect the hospital-level congestion. The result showed that the critical care demands remained to be significantly associated with the outcome (Supplementary Table 2).

**Table 2:** Association between the overall medical demands and mortality

Factor	ORs	*p* value
Age	1.07 (1.06–1.08)	<0.001
Sex, male	0.99 (0.88–1.12)	0.87
BMI (ref. < 25)		
25 ≤ BMI < 30	0.90 (0.79–1.01)	0.083
BMI ≥ 30	0.97 (0.82–1.14)	0.71
Use of ECMO	4.42 (3.77–5.19)	<0.001
Use of NIV or HFNC before MV	1.38 (1.22–1.55)	<0.001
Pandemic wave (ref. second wave)		
Third wave	1.32 (1.04–1.68)	0.022
Fourth wave	1.27 (0.99–1.63)	0.055
Fifth wave	1.67 (1.28–2.17)	<0.001
Sixth wave	1.16 (0.83–1.63)	0.38
Seventh wave	0.92 (0.51–1.65)	0.78
Eighth wave	1.24 (0.78–1.95)	0.36
Overall medical demands	1.00 (1.00–1.01)	0.63

*BMI* body mass index, *ECMO* extracorporeal membrane oxygenation, *NIV* non-invasive ventilation, *HFNC* high-flow nasal cannula, *MV* mechanical ventilation, *OR* odds ratio

**Table 3:** Association between the critical care demands and mortality

Factor	ORs	*p* value
Age	1.07 (1.06–1.08)	<0.001
Sex, male	0.99 (0.88–1.12)	0.89
BMI (ref. < 25)		
25 ≤ BMI < 30	0.90 (0.80–1.02)	0.11
BMI ≥ 30	0.98 (0.83–1.16)	0.81
Use of ECMO	4.51 (3.84–5.30)	<0.001
Use of NIV or HFNC before MV	1.35 (1.20–1.52)	<0.001
Pandemic wave (ref. second wave)		
Third wave	1.08 (0.83–1.39)	0.57
Fourth wave	0.94 (0.71–1.23)	0.63
Fifth wave	1.10 (0.81–1.50)	0.54
Sixth wave	0.98 (0.73–1.31)	0.88
Seventh wave	0.91 (0.63–1.29)	0.58
Eighth wave	1.18 (0.84–1.66)	0.35
Critical care demands	1.11 (1.07–1.16)	<0.001

*BMI* body mass index, *ECMO* extracorporeal membrane oxygenation, *NIV* non-invasive ventilation, *HFNC* high-flow nasal cannula, *MV* mechanical ventilation, *OR* odds ratio

In addition, multiple imputation was performed on the 11,289 eligible patients before excluding cases with missing data (imputation method: predictive mean matching for continuous variable (age), logistic regression model for binary variables (death, gender and HFNC or NPPV before intubation) and polytomous logistic regression model for categorical variable (BMI), number of datasets: 20, number of iterations: 50). A multivariable logistic regression was conducted using the imputed data, and as in the primary analysis, critical care demands were identified as a significant factor that increased mortality (Supplementary Table 3).

### Effect of critical care demands on the mortality in relation to the average mortality of the hospital (relationships of variations in critical care demands and baseline risk across hospitals)

**Fig. 3:**
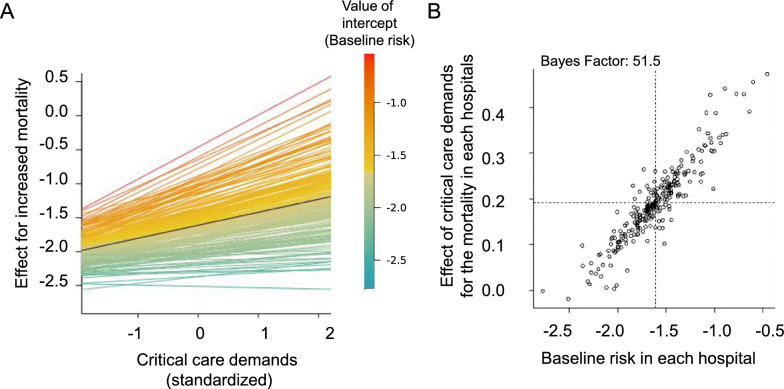
Impact of critical care demands among hospitals with different baseline risks. Analysis using Bayesian estimation with a mixed-effects model considering the variability in baseline risk and the variability in the impact of critical care demands among hospitals were performed. **A** Horizontal axis shows the standardized critical care demands, the vertical axis shows the effect on the increased mortality. Each line represents the predicted effect of critical care demands on the mortality at each hospital. The color of the line represents the baseline risk, which was calculated from the random effect of the intercept grouped by hospitals. **B** Scatter plots showing the baseline risk at each hospital on the horizontal axis, hospital-specific effect of critical care demands on the mortality on the vertical axis. The shape of the density distribution reflects the correlation between the effect of critical care demands on the mortality and the baseline risk

We also performed an exploratory analysis using another generalized linear mixed-effects model, taking into account the variability in baseline risk among hospitals and the variability in the impact of the national critical care demands among hospitals. The estimated correlation coefficient between the baseline risk of a hospital and the critical care demands was 0.67 (95% credible interval: 0.30–0.99) (Fig. 3). In addition, we compared a model in which the effect of the critical care demands was independent from baseline risk with another model in which the two effects were positively correlated, which yielded a Bayes Factor of 51.5. This represented strong evidence of a positive correlation of random effects, that is, that the effect of the national critical care demands on the mortality was more pronounced in hospitals with higher baseline risk.

## Discussion

There were no studies to investigate the association between increased medical demands nationwide and the mortality of COVID-19 patients requiring mechanical ventilation. In the present study, we successfully demonstrated (1) that the critical care demands, but not the overall medical demands at the country level, were associated with the mortality of mechanically ventilated patients with COVID-19 and (2) that the effect of the national critical care demands on the mortality was higher in hospitals with higher baseline risk.

Our findings lend support to the importance of counting the number of severe COVID-19 patients rather than the number of newly confirmed COVID-19 patients for evaluating the risk of mortality of severe COVID-19 patients in a country. Since the number of newly confirmed COVID-19 patients may not be a reliable indicator of healthcare burden, as it can be strongly influenced by the accessibility to diagnostic tests and COVID-19 patients do not always require hospitalization, it may not accurately reflect the degree of critical care medical demands for treating severe COVID-19 patients. On the other hand, our spline curve showing an almost linear relationship between the critical care demands and the mortality suggest that it may be beneficial to try, as a national strategy, to reduce the number of critically ill patients per day as much as possible, which could potentially contribute to an overall decrease in their mortality rate.

This is the first study conducted to evaluate the effect of increased demands for medical resources by patients with severe COVID-19 in Japan. It should be noted that Japan did not experience a devastating medical resource crisis; our national survey revealed that a shortage of mechanical ventilators/other equipment and medications actually occurred in less than 20% of the facilities, and that non-ICU medical staff provided support in the ICU in approximately 50% of the surveyed facilities [25]. Therefore, our findings may suggest the existence of reasons beyond shortage of medical equipment for the increased mortality, such as alterations in the treatment strategies adopted by the medical staff on account of fear of a potential shortage of medical equipment, or decreased quality of treatment provided in the ICU due to the shortage of ICU specialists. However, further study is needed for a better understanding of the possible impact of these issues.

Our analyses using a generalized linear mixed-effects model accounting for inter-facility differences identified a positive correlation ratio of 0.67, which can be regarded as representing a strong correlation [26] between the random effect of critical care demands on the mortality and the baseline risk of hospital. Our results suggest that hospitals with higher baseline risk may have been less “elastic” in the face of a surge of severe COVID-19 patients as compared with those with lower baseline risk, which was consistent with the results of studies focused on non-COVID patients prior to the pandemic [5]. Although this was an exploratory analysis, the Bayes factor of 51.5, obtained from comparing models where random effects are independent versus models where random effects are positively correlated, strongly suggests the existence of this correlation. Our results may suggest that the hospitals accepting the patients with a predicted higher mortality should be prioritized for managing reallocation and distribution of the limited medical resources available during a pandemic. Incorporating baseline risk into resource allocation strategies could guide policymakers in more efficiently directing resources to hospitals under greater strain, ultimately improving patient outcomes. This approach could also serve as the foundation for developing dynamic, real-time resource allocation systems that respond to fluctuations in critical care demands during future pandemics, thereby enhancing healthcare system resilience and reducing mortality.

This study had several limitations. First, it was a retrospective observational study, and there may have been some confounding factors not included in our national database that could have substantially influenced the outcome. We attempted to adjust in this study as much as possible by using several factors available from CRISIS database, such as the use of ECMO and the use of non-invasive respiratory support before intubation (we considered that can reflect the patient severity). However, factors such as viral strain variations, vaccination status, and the use of antiviral or neutralizing antibody therapies were not accounted for. These factors could have influenced patient prognosis and should be considered in future studies for a more comprehensive analysis. Second, the definition of severe COVID-19 adopted by the Japanese MHLW was slightly different from that in CRISIS, because the MHLW’s definition included patients who were ventilated, on ECMO, or treated in the ICU. Third, while we mainly considered medical demands, we did not examine medical supplies in detail. The supply of medical care involves not only ICU beds, but also the number of staff, equipment, and regional variations in healthcare infrastructure and resource distribution, all of which can significantly impact patient outcomes. Due to the practical difficulty of collecting such detailed supply-side information daily across different regions, we focused on analyzing the impact of increasing medical demand in this study. However, we acknowledge that future research should include a more comprehensive analysis that balances both supply and demand factors, taking into account regional differences in resource availability. Fourth, further investigation is needed to identify the precise reason for the strong relationship between the effect of national critical care demands on the mortality and baseline risk of a hospital, which remains unknown. Fifth, a considerable amount of missing data, including outcomes, was observed. In particular, there is a possibility of selection bias, as cases transferred during the acute phase may exhibit higher severity. However, even in the analysis using data imputed through the multiple imputation method, critical care demand remained a significant factor associated with mortality. Sixth, we used the nationwide number of newly confirmed cases and severe COVID-19 patients on the day of intubation as indices. While these numbers could fluctuate during ICU treatment, we focused on the intubation day, because it represents a critical turning point in the treatment process. Strain on medical resources at that time can significantly impact care quality. Although it would be ideal to account for fluctuations throughout the hospital stay, we decided to assess resource strain at the time of intubation because of practical limitations in data collection and analysis and the unpredictability of future patient numbers. Seventh, hospital-specific conditions may not be fully accounted for. In addition to adjusting for inter-facility differences using random effects, we also conducted sensitivity analyses that took into account hospital-level congestion. These analyses ensure that our findings are robust, considering both national-level data and hospital-specific conditions. However, this approach may not fully capture certain detailed medical factors, such as the detailed congestion level and number of staff at each hospital, as well as non-medical factors like hospital policies, triage protocols, and decision-making processes. These factors could have influenced patient outcomes and resource allocation during critical care surges, and their impact should be considered in future studies. Finally, the management of COVID-19 during the pandemic differed among countries [27], which could have an influence on the association between a surge of patients and the outcome. We need to evaluate the trend we observed in Japan was also observed in other countries.

## Conclusions

The national critical care demands, but not overall medical demands, were independently associated with the mortality of COVID-19 patients requiring mechanical ventilation in Japan. This effect was more pronounced in hospitals with higher baseline risk as compared with hospitals with lower baseline risk. Our results may be helpful for constructing a better national strategy for allocating and distributing medical resources during pandemics of severe respiratory diseases.

## Supplementary Information

**Additional file 1. Supplementary Figure 1.** Flow diagram of patients.

**Additional file 2.**

**Additional file 3.**

**Additional file 4.**

## Data Availability

The data set analyzed in this study is not publicly available due to contractual agreements with the hospitals that provided the data to the database.

## Abbreviations

COVID-19: Coronavirus disease 2019
ICU: Intensive care unit
CRISIS: CRoss Icu Searchable Information System
ECMO: Extracorporeal membrane oxygenation
BMI: Body mass index
NIV: Non-invasive ventilation
HFNC: High-flow nasal cannula
MV: Mechanical ventilation
OR: Odds ratio
