# Supplementary material for: Increased national critical care demands were associated with a higher mortality of intubated COVID-19 patients in Japan: a retrospective observational study
Source: J Intensive Care. 2024 Nov 5;12:46. doi: 10.1186/s40560-024-00758-8 (PMC11536903; doi:10.1186/s40560-024-00758-8)
Supplement: Supplementary file 1 — Additional file 1. Supplementary Figure 1. Flow diagram of patients. [file 40560_2024_758_MOESM1_ESM.pptx]

## Slide 1
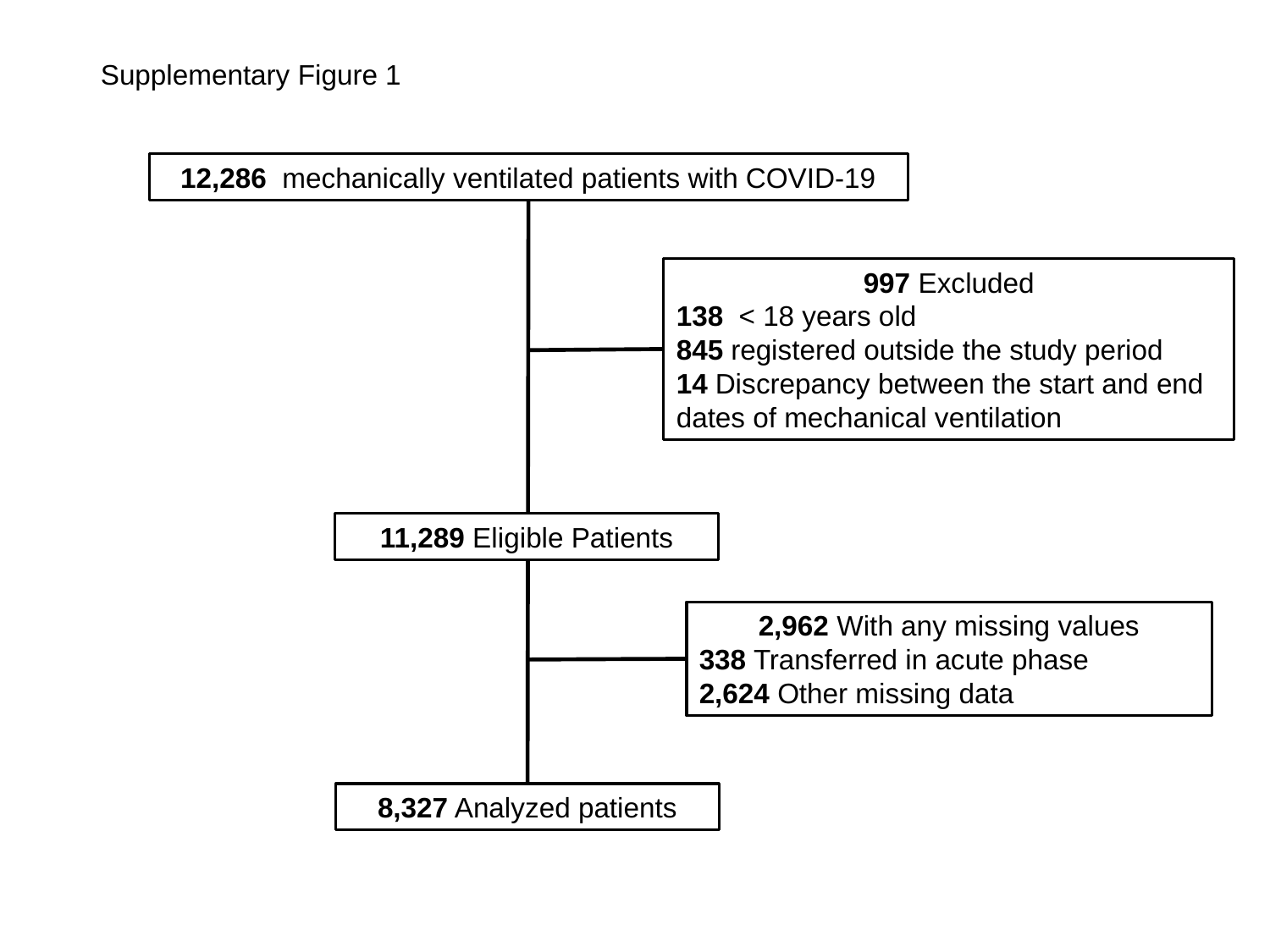

Supplementary Figure 1
12,286 mechanically ventilated patients with COVID-19
997 Excluded
138 < 18 years old
845 registered outside the study period
14 Discrepancy between the start and end dates of mechanical ventilation
11,289 Eligible Patients
2,962 With any missing values
338 Transferred in acute phase
2,624 Other missing data
8,327 Analyzed patients
